# Synthesis of metal–organic framework functionalized macroscopic flow-through precipitate tubes

**DOI:** 10.1038/s41598-025-97630-y

**Published:** 2025-04-17

**Authors:** Edina Balog, Kinga Bene, Gábor Schuszter

**Affiliations:** Department of Physical Chemistry and Materials Science, University of Szeged, Rerrich Béla tér 1., Szeged, 6720 Hungary

**Keywords:** Materials chemistry, Physical chemistry

## Abstract

The guided growth and the composition control of the well-known chemical garden tubular structures have been widely studied in the literature. However, the applicability of these macroscopic hollow precipitate tubes (e.g., for catalysis, sensorics etc.) is still limited, since these pipes originally do not have a flow-through character, thus the functionalization of these tubes is difficult to implement. In this work, our goal was to design a novel reactor that enables the production of these flow-through precipitate pipes with robust junctions, and thus their functionalization for further applications. We successfully built the reactor and synthesized such pipes. Their flow-through character was proven in case of various template tubes which were produced by injecting one of the reactant solutions into the pool of the other in three dimensions. After the production of the template tubes, we attempted to decorate the surface with sodalite type ZIF-8 crystals, which are of great interest thanks to their beneficial properties (porous structure, huge specific surface area etc.) for catalysis or gas separation. The surface functionalization was carried out by exchanging the reactant solutions inside and outside the template precipitate tubes. Due to the semi-permeable nature of the tube wall, the reactants could diffuse through the membrane and react with each other. This way we produced (most probably sodalite type) ZIF-8 crystals on the inner tube surface and thus functionalized it.

## Introduction

The chemical garden phenomenon is a well-known example of spatial structures formed in nonequilibrium systems. During the traditional experiment, various transition metal salt seeds are added to a sodium silicate aqueous solution, thus a metal–silicate precipitate membrane can form at the interface between the two reactants, which acts as a semi-permeable membrane. Via osmosis, the water molecules flow into the precipitate membrane from the sodium silicate solution outside, which increases the pressure inside the diaphragm, thus it splits. In this way, the solution containing the metal cations flows out and can react with the silicate anions, which results in the formation of a new semi-permeable membrane. Beside the osmotic effect, buoyancy caused by the density difference between the solutions also plays an important role, thus the upward growth of the precipitate tubes can be observed in the vessel^1^. Numerous examples have been presented in the literature for the abundance of these three dimensional precipitate tubes. For example, the structures emerging on a metal surface during corrosion are similar to chemical gardens^2^. The mechanisms of the growth of silicate gardens and of the hydration of Portland cement are also analogous, thus we can obtain information about the structural properties of cement^3^. In the case of polyoxometalate tubes, the controlled growth would pave the way for catalytic applications^4^. In order to exploit this, it became necessary to produce (chemical garden-like) precipitate tube structures in a more controlled manner.

The production of precipitate tubes coupled to various transport phenomena (e.g., osmosis or flow) is a promising method for designed synthesis, which is widely studied in the literature^5–9^. An important aspect is that various spatial gradients (e.g., concentration and *p*H) emerge in these systems. By the external control of these gradients, the micro- and macrostructure of the forming products (e.g., morphology, crystal size, composition, and tube diameter) can be influenced^10,11^. For example, by changing the experimental parameters (e.g., reactant concentrations, stoichiometric ratio, flow rate etc.), the dynamic behavior of the systems can be altered, which makes the production of arbitrary structures feasible^12^. Furthermore, by using flow-driven systems, precipitate tubes with different internal and external microstructures can be yielded, which supports injection methods^13^. Although the production of precipitate tubes by injection process can be carried out in one-, two, or three-dimensions^1,14,15^, the majority of 3D tubes are obtained by relying on the traditional chemical garden phenomenon^16^. Beside the simple injection process, various methods have been presented in the literature to influence the structure and composition of the forming product, e.g., bubble guidance^17^, magnetic-field manipulation^18^, and peristalticity-driven techniques^19^. A common property of the 3D tubes produced by the above methods is their hollow pipe-like structure. Therefore, in principle, they could be used as flow-through reactors. For example, if a precipitate tube has a catalytic feature, it is possible to adsorb different reagents on its surface, thereby it allows further reactions in a gradient field^20,21^. It is also possible to produce precipitate tube with incorporated quantum dots as functionalities. Steinbock et al. found that these semiconductor nanoparticles were chemically accessible, thus these tubes could be suitable tools for sensory applications^22^.

However, the precipitate tubes presented so far in the literature are either inherently closed on one end by the end of the synthesis, and thus they do not have a flow-through character, or are open at the ends but without any possibility to connect them to any junctions. In one specific example, when injected solution (and only that) was exchanged during the experiment, and authors claimed they produced flow-through precipitate tubes, the structures were heavily branched and closed on the growing tips at the end of injection. Therefore, these tubes without robust junctions at the ends were not applicable as flow-through reactors^23^. As a result, the proposed widespread application of these hollow tubes, such as catalysis^24^, microfluidics^25^, sensorics^22^ etc., is not possible; they cannot be installed as part of a reactor setup. Therefore, in terms of applicability, it has become essential to develop a method that enables the production of flow-through precipitate pipes.

Accordingly, in the present work our goal was to design an experimental setup and elaborate the corresponding protocol, which enable the production and functionalization of a flow-through hollow precipitate tube. We attempted to produce a pipe with some potentially useful feature, therefore metal–organic framework (MOF) precipitate tubes were fabricated in the reactor. MOFs are crystalline materials and they are consisting of metal ions (e.g., Zn(II) or Co(II)) coordinated to organic ligands (e.g., oxalic acid or 2-methylimidazole) to form one-, two-, or three-dimensional structures. ZIF-8 type MOFs are composed of Zn(II) ions connected by 2-methylimidazole (2-MeIm) linkers, while in the case of ZIF-67 system, we apply Co(II) ions instead of Zn(II) ions. These materials have extremely porous structure and thus remarkable specific surface area^26,27^. Due to these advantageous properties, they are used in various fields of chemical technology, for example as catalysts or adsorbents^28–31^. If these crystals build up a flow-through precipitate tube, the visioned applications become achievable. Various polymorphs of ZIF-8 are known in the literature, which have different thermodynamic stability and porosity. Due to its porous structure, the sodalite-like ZIF-8 polymorph (SOD) has a large specific surface area, but its formation is thermodynamically less favorable compared to other polymorphs (e.g. *dia*(Zn))^32^. In our previous work we pinpointed that, by applying relatively high reactant concentrations, we can achieve a kinetic control over product properties in a well-stirred system, and the thermodynamically less stable but useful polymorph of ZIF-8 (SOD) can form as the final product^33^. Furthermore, the excess of linker molecules improves the crystallinity of the product ([2-MeIm] : [Zn(II)] = 20 : 1) according to the literature^34^. The addition of base is also necessary in order to ensure the presence of 2-methylimidazolate ion (2-MeIm$^-$), which favors the formation of tetrahedrally coordinated crystal structure^35^. Taking all these aspects into account, in the current work we attempted to produce sodalite-like ZIF-8 crystals on the inner surface of flow-through precipitate tubes.

**Fig. 1:**
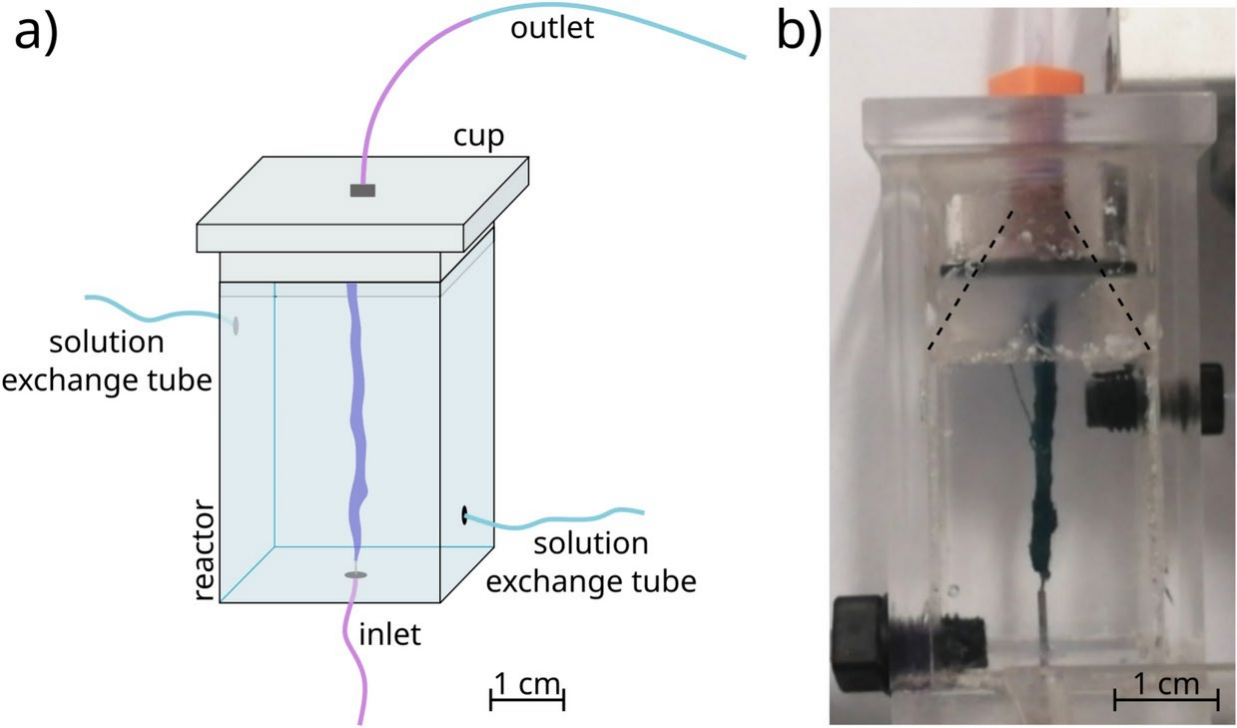
(**a**) Schematics and (**b**) realization of the experimental setup: vertical 3-dimensional Plexiglas reactor containing a well-grown flow-through precipitate tube which then serves as a template for functionalization. The solution exchange tubes are for functionalisation of the formed precipitate tube. The dashed lines lead the eye along the conic shape of the reactor cup.

## Results and discussion

In this work our main goal was to design a reactor which enables the production and functionalization of flow-through precipitate tubes. During the synthesis of such precipitate tubes, one of the reactant solutions was filled into the reactor and the other one was injected from below into the host solution by using a syringe pump (Fig. 1a). In order to facilitate the proper upward growth of the pipes, we applied two technical inventions. First, the reactor body was closed by a cup whose interior had a conic shape ending in an outlet tube (Fig. 1b). This upward narrowing cone drove the growing precipitate tube towards the outlet. The second technical invention was that, simultaneously with the injection from below, the reactor vessel was continuously emptied from above through an outlet by a syringe pump in withdrawal mode (Fig. 1a). The flow-through character of the grown precipitate tube was proven by the color of the injected solution which appeared in the outlet pipe (see Fig. 1a for schematics, and Fig. S3 for experimental evidence). In order to functionalize the tube wall, we designed the reactor in such a way that it is suitable for exchanging the solutions inside and around the tube. In favor of the replacement of the external solution, we made two more connections to the reactor batch (Fig. 1a). The exchange of the internal solution was carried out by injecting the replacement solution from the same inlet as before. Cross diffusion of ionic constituents through the membrane made possible to decorate the precipitate wall with the desired crystals. More detailed description of the operation is included in the “Methods” section, and a step-by-step chart is presented in SI (Fig. S2). In general, the obtained precipitate tubes are ca. 5 cm long, their typical inner diameter is 1–3 mm, and the wall thickness is ca. 15–20 μm. All these properties can be tuned by the experimental parameters (e.g., flow rate, injection port size, time of cross diffusion etc.), but this does not fall into the scope of our study.

**Fig. 2:**
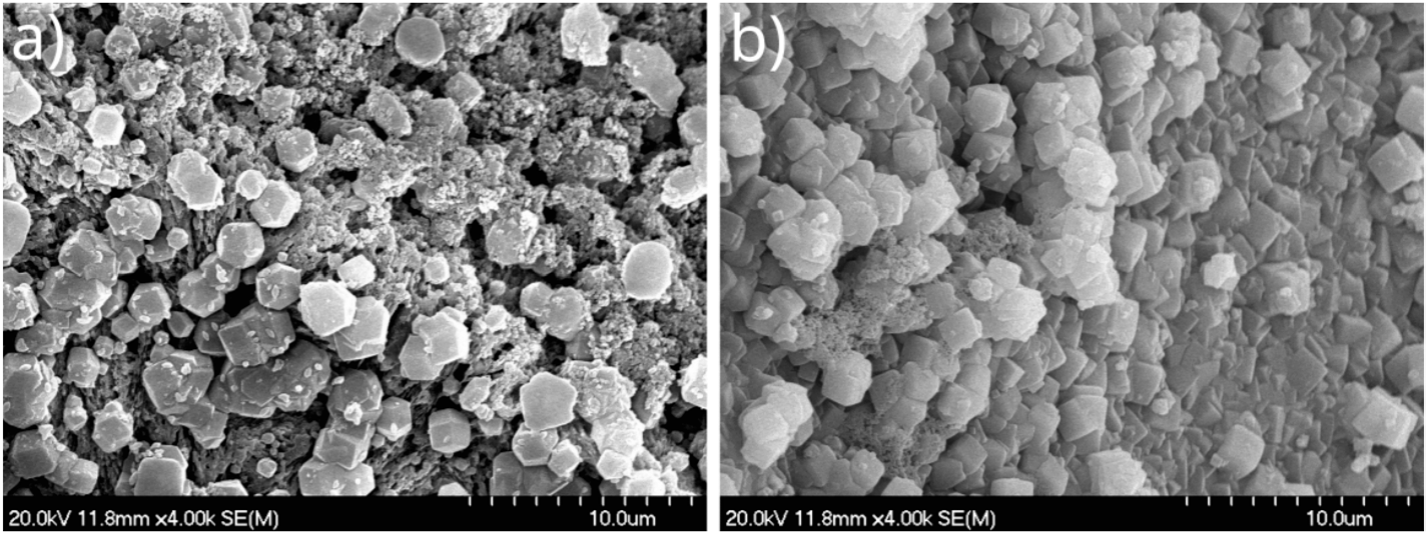
(**a**) Outer and (**b**) inner surface of a zinc–silicate precipitate tube with crystals on it whose shape is indicative of SOD ZIF-8, which were produced by changing the silicate solution to 2-MeIm solution outside the tube.

As a reference experiment, we attempted to synthesize a precipitate tube purely made of ZIF-8 due to the advantageous properties of this material^26,27^. During the experiments, 0.05 M ZnCl$_2$ aqueous solution was injected into 1 M 2-methylimidazole (2-MeIm) solution, which was alkalized earlier (0.1 M NaOH). These reactant concentrations provided us with high quality ZIF-8 (SOD) crystals in our previous work carried out in a well-stirred system^33^. In the present injection experiments, during the emergence of ZIF-8 precipitate, we observed that the tubular structure could not be obtained at the applied experimental parameters. Therefore, in the next step, we tried to create a ZIF-8 containing tube by producing a template tube first, and then synthesizing SOD ZIF-8 crystals on its surface via solution exchanges and cross diffusion. Under appropriate conditions Zn(II) and silicate ions react while providing a precipitate tube^36^. In this tube oxide and oxo-hydroxide can be also present in a large amount beside the silicate^37^. Therefore, during our experiment, this template tube was created by pumping 0.5 M Zn(II) solution into 0.75 M Na$_2$SiO$_3$ solution with 1 ml/h volumetric flow rate for 45 min. The obtained tubular structure had a flow-through character, i.e., it was a proper pipe. This already demonstrates the applicable design of our new reactor configuration. In order to produce SOD ZIF-8 crystals on the inner surface of this zinc–silicate pipe, we only had to change the outer silicate solution due to the presence of Zn(II) inside the tube. Therefore, the external solution was swapped to 1 M 2-MeIm solution which contained 0.1 M NaOH as well, while the ZnCl$_2$ solution flowed continuously inside the tube walls for 3 h with 4 ml/h flow rate. At the end of the process, the tube was flushed with distilled water inside and outside in favor of preventing further reactions. Then the tube was removed from the reactor, and its microstructure analysis was carried out by using scanning electron microscopy (SEM) and energy-dispersive X-ray spectroscopy (EDX). More experimental details can be found in the “Methods” section. Images were taken from the outer (Fig. 2a) and inner (Fig. 2b) surface of the tube, as well. By knowing the reactants present in the system, the shape of crystals can be used as a first indication to whether or not SOD ZIF-8s are produced, since they exhibit a hexagonal particle shape which is clearly not the case for the silicate material of template tube.

Therefore, according to the shape of crystals, both the inner and outer surfaces are possibly covered with the SOD polymorph of ZIF-8 crystals, which is the positive result due to its advantageous properties. The presence of these crystals is further supported by detection of Zn and C atoms outside (C: 26.4 ± 2.8 at%, Zn: 7.5 ± 2.6 at%) and inside (C: 12.7 ± 1.2 at%, Zn: 11.1 ± 3.2 at%) the tube, which are involved in the structure of ZIF-8 materials, but not in a traditional silicate garden tube. At this stage of our study we did not investigate more the composition of the products. Crystal shapes and EDX mapping were used as first indications to gain information about a possibly successful synthesis of the SOD ZIF-8 decorated precipitate tube. This way we reduced the time required to judge whether the synthesis protocol was likely appropriate or not. Once the appropriate protocol was found, the final precipitate structure was thoroughly investigate which will be introduced later.

**Fig. 3:**
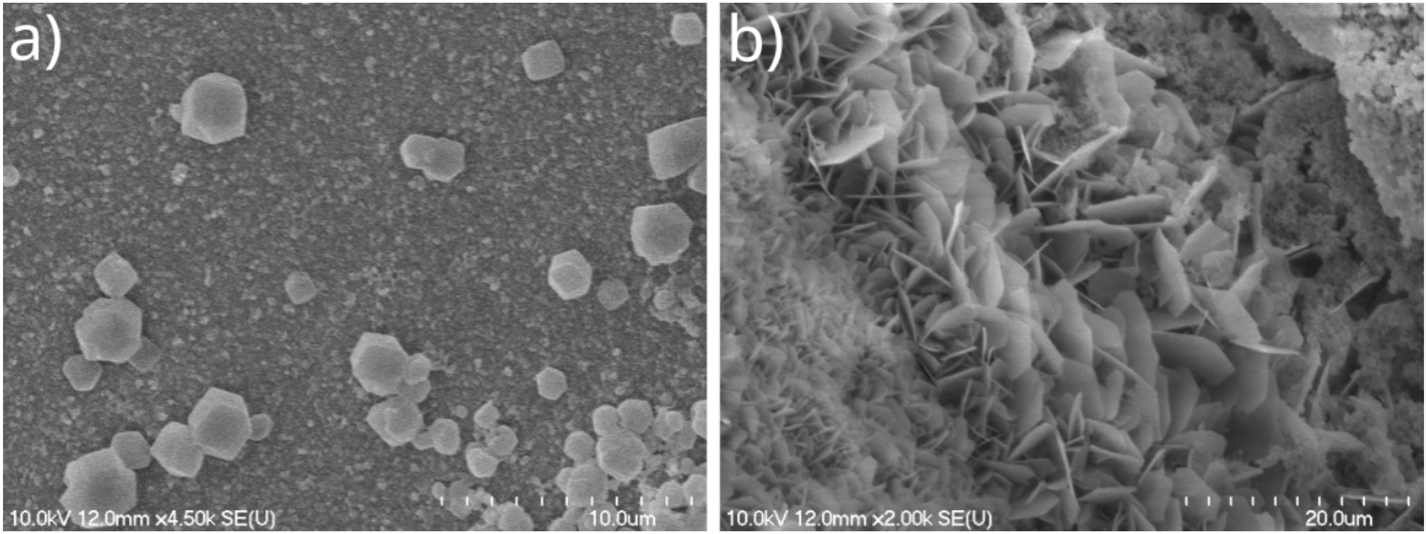
(**a**) Outer and (**b**) inner surface of a cobalt–silicate precipitate tube with ZIF-8 crystals on it, which were produced upon solution exchanges.

As seen above, by applying the zinc–silicate template tube, we observed that the SOD polymorph likely forms inside and outside the tube surface as well. However, the formation of SOD is probably caused by the dissolution of zinc silicate in 2-MeIm solution because of the difference in solubility products, thus 2-MeIm may take the place of silicate in the precipitate tube. As a consequence, this can lead to the collapse of the precipitate tube after a longer time of solution exchange, because we previously observed that the ZIF-8 precipitate does not yield a tubular structure. Due to this disadvantage, the further application of this type of ZIF-8 containing tube is not preferred. Therefore, we decided to apply such template tubes which do not contain any of the original reactants, i.e., neither zinc nor 2-MeIm. Consequently, in the next step, we attempted to decorate other types of template tubes with SOD ZIF-8 crystals by changing the solution outside and inside the tube. In this case, the cross diffusion of reactant ions through the precipitate wall, caused by the concentration gradient between the two sides, can solely lead to the formation of the desired crystals on its surface. During our preliminary experiments we established that cobalt silicate precipitate tube has a great mechanical and pH stability in neutral and alkaline range. Due to this we have chosen cobalt silicate as a template tube, which was produced by flowing 0.5 M CoCl$_2$ aqueous solution with 1 ml/h flow rate into a pool of 0.75 M Na$_2$SiO$_3$ solution. After we successfully grew the precipitate tube, and made sure that it has a flow-through character (appearance of the color of the injected solution in the outlet tube in Fig. 1a), we attempted to decorate its surface with SOD ZIF-8 crystals. At first, we replaced the external silicate solution by 1 M 2-MeIm solution. These experiments were performed without the addition of any alkaline chemical, because we showed earlier in a well stirred system, that it is not essential for the formation of the SOD polymorph of ZIF-8^33^. In order to check whether the tube remains intact after the solution exchange, we restarted the pumping of CoCl$_2$ solution inside and observed it passing through the tube. This again highlights that the newly proposed reactor configuration works properly, and facilitates the solution exchange both inside and outside the already grown precipitate tube. In the next step we swapped the solution inside the tube to 0.05 M ZnCl$_2$, which was injected with 0.4 ml/h flow rate for 1.5 hours to provide enough time for the crystal growth. At the end of the experiment, distilled water was flowed inside the precipitate tube for 45 minutes at 1 ml/h flow rate to flush it. After that, the external solution was also replaced by distilled water for proper rinsing. Finally, the microstructure and composition of the samples taken from the precipitate tubes were examined using SEM and EDX. According to the SEM images, small crystals are present outside the tube which resemble to SOD ZIF-8 crystals published in the literature (Fig. 3a). In contrast, the interior surface of the tube is covered with plate-like crystals which are similar to the *dia*(Zn) polymorph of ZIF-8 crystals (Fig. 3b)^32^. The composition analysis also indicated the presence of ZIF-8 crystals on the outer surface, as the amount of carbon is significant (C: 58.6 ± 18.0 at%), which proves the incorporation of imidazole. In case of the crystals present on the inner surface, zinc can be detected (Zn: 6.8 ± 1.6 at%), however, the amount of carbon is significantly smaller (C: 7.5 ± 0.6 at%) compared to the outer surface. The reason for this is probably that 2-MeIm cannot diffuse into the tube in adequate amount. This could be the cause for the presence of the unwanted DIA ZIF-8 inside the tube (Fig. 3b), since 2-MeIm is not present in a sufficient stoichiometric excess to form SOD ZIF-8.

**Fig. 4:**
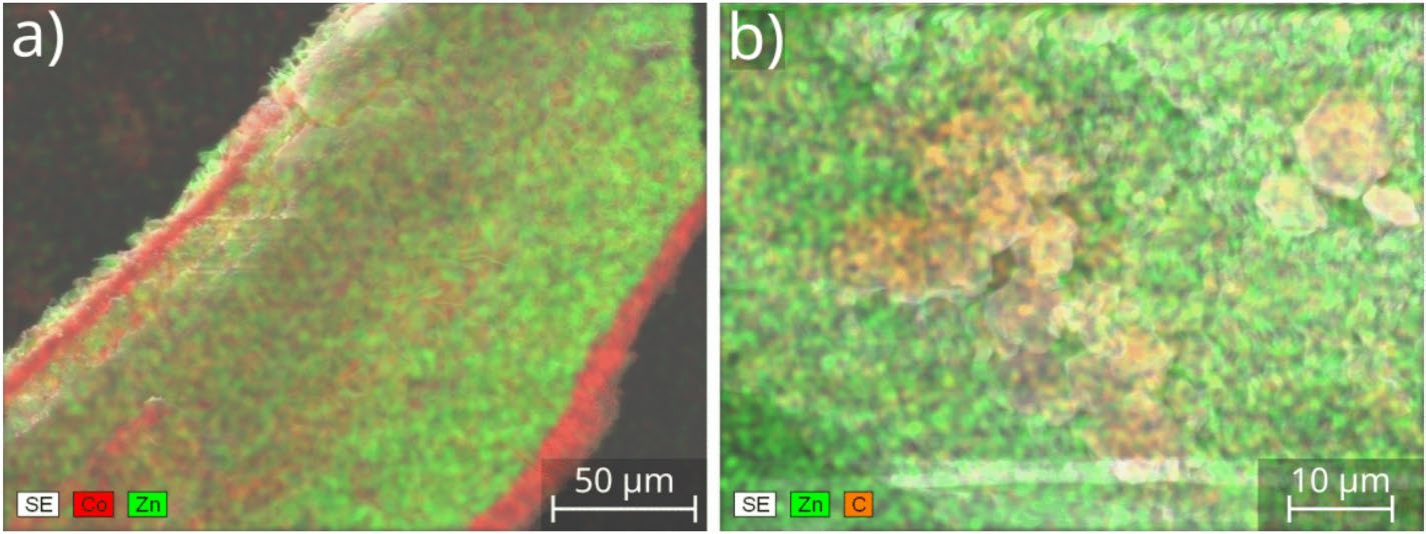
Artificially colored elemental map of the inner surface of a cobalt–silicate precipitate tube after the outer sodium silicate solution was replaced by 2-MeIm, and the inner CoCl$_2$ solution by ZnCl$_2$; illustration of (**a**) Co/Zn and (**b**) Zn/C ratio.

Therefore, to improve the functionalization of the precipitate tube, i.e., in order to precipitate SOD ZIF-8 crystals inside as well, further experiments were performed. During these experiments the cobalt silicate tube was produced in the same way as previously (${\hbox {c}_{\text{CoCl}}}_2$ = 0.5 M, ${\hbox {c}_{\text{Na}}}_2 \text{SiO}_3$ = 0.75 M, Q = 1 ml/h, t = 45 min), but now the 1 M 2-MeIm used for solution exchange contained 0.1 M NaOH additionally (*p*H$\sim$13), because it is known to favor deprotonation of 2-MeIm, and thus may promote the formation of SOD. This base concentration is not high enough to trigger the hydrolysis of 2-MeIm within the time frame of the experiment^33^. After the exchange of the external solution, ZnCl$_2$ solution was injected inside with 0.4 ml/h flow rate for 1.5 hours, and then the outside and inside of the precipitate tube were washed with distilled water as mentioned previously. Finally, we took samples from the precipitate for EDX analysis. Figure 4a shows the inner part of the tube, and its outer surface is also moderately visible (two red bands on both edges of the tube wall). It was observed that, the exterior part and the bulk of the tube remained the original cobalt-containing material. However, the inner surface is mostly composed of zinc (Zn: 11.2 ± 3.3 at%), although cobalt can also be detected in small amount (Co: 3.8 ± 1.1 at%), since the template tube was cobalt silicate. It is known from the literature that Co(II) can also form MOF structure with 2-MeIm organic ligand (ZIF-67), thus in order to investigate whether the detectable cobalt is present in the form of cobalt silicate precipitate or ZIF-67, we studied more closely the crystals on the inner surface. Figure 4b shows that, according to the shape of the crystals, possibly the high porosity ZIF-8 polymorph (SOD) was present inside the tube (small hexagonal crystals marked with orange in Fig. 4b). However, no significant zinc was found in these crystals, which suggests that these could be the cobalt containing SOD ZIF-67. The presence of ZIF-67 instead of cobalt silicate on the surface is proven by the presence of carbon in these crystals. According to these results, the zinc-containing ZIF-8 was not formed during these experiments, which can probably be explained by the large size of 2-MeIm, which hinders the diffusion of this molecules through the pores of the tube. We assume that 2-MeIm reached the inner part of the tube by partially dissolving the cobalt–silicate membrane, thus taking the place of the silicate in the tube. In the next few experiments, the production of SOD ZIF-8 crystals inside the precipitate tube was further attempted. At first we replaced the solutions inside and outside the cobalt silicate tube again, but now the precipitate tube was left to soak in the alkalized 2-MeIm external solution for significantly longer time ($\approx$18 h), thereby allowing more time for the diffusion of 2-MeIm molecules. Beside this, we also performed experiments in which the ZnCl$_2$ solution was flowed inside the tube for a longer time (3 h instead of 1.5 h). However, none of these methods helped to precipitate SOD ZIF-8 crystals on the inner surface of the tube.

**Fig. 5:**
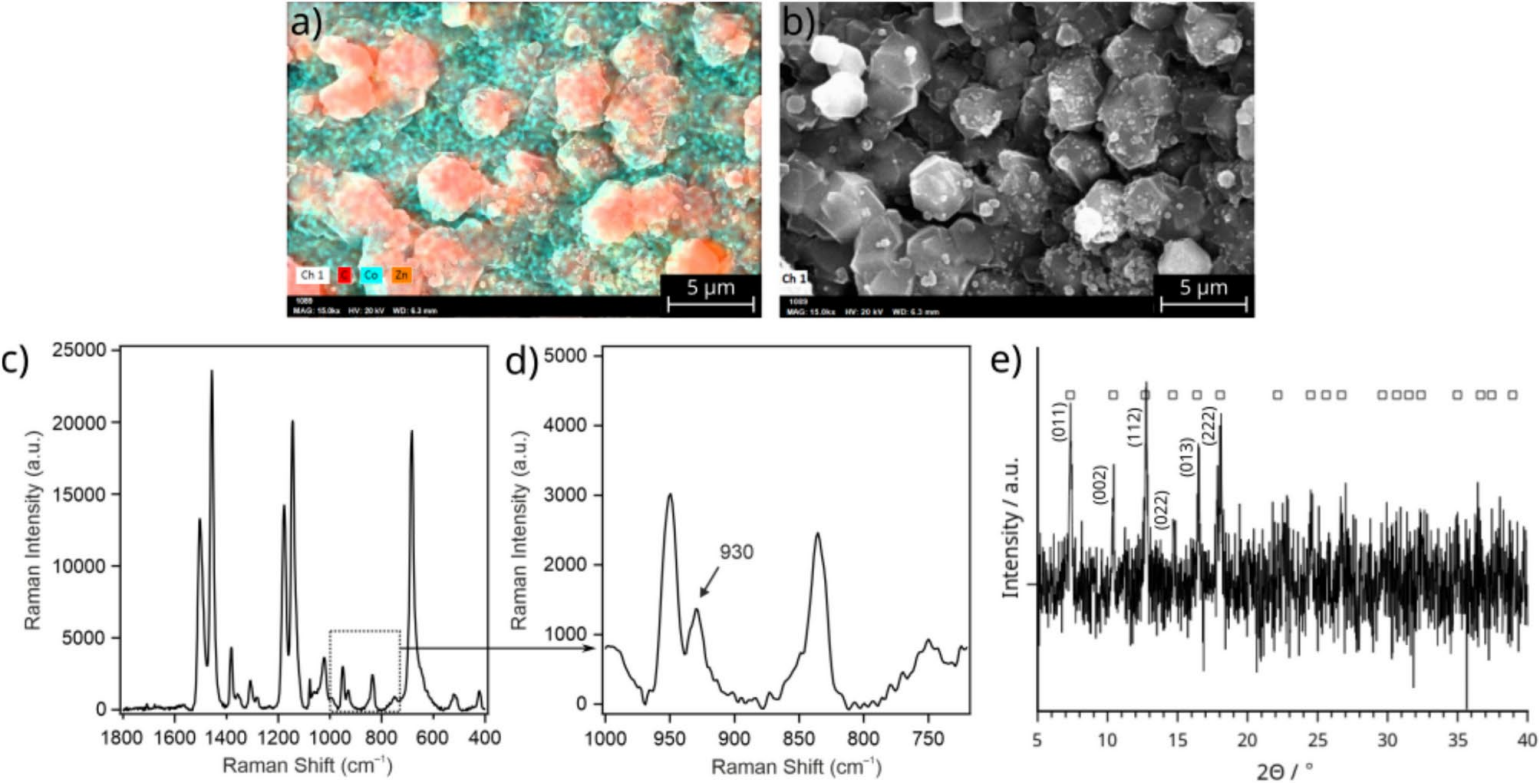
(**a**) EDX, (**b**) SEM, (**c**, **d**) Raman microscopy, and (**e**) powder X-Ray diffraction analysis of the inner surface of a cobalt–silicate precipitate tube after the outer sodium silicate solution was replaced by ZnCl$_2$, and the inner CoCl$_2$ solution by 2-MeIm. For the PXRD measurements the 2-MeIm solution was injected for 16 hours; background corrected diffractogram. The artificially colored elemental map (**a**) illustrates the distribution of C/Co/Zn (**d**) is a close-up of the Raman spectrum shown in (**c**). Squares above the diffractogram (**e**) belong to SOD ZIF-8 diffractions^38,39^.

Finally, the decoration of cobalt–silicate tube with SOD ZIF-8 crystals was attempted by reversing the replacement solutions, i.e., the external silicate solution was replaced by ZnCl$_2$ (0.5 M), and the internal Co(II) solution by alkalized 2-MeIm (0.5 M 2-MeIm and 0.1 M NaOH). To do so, the template tube was produced as previously (injection of 0.5 M CoCl$_2$ into 0.75 M Na$_2$SiO$_3$). However, before the solution exchanges, we first replaced both the internal and external solutions by distilled water. This prevented the formation of unwanted products, because the Co(II) ion originally present inside the tube could precipitate with the newly injected 2-MeIm. In addition, the Na$_2$SiO$_3$ which formerly surrounded the tube could also react with the replacement Zn(II) solution. Distilled water was flowed for 1 h inside the tube. When the flushing was finished, we filled the ZnCl$_2$ solution around the tube and then we could start the injection of the alkalized 2-MeIm solution into the tube at a flow rate of 0.4 ml/h. After 3 h injection, the inner and outer solutions were replaced by water again. The composition of the tube was first studied by EDX. It was found that the crystals which form inside the precipitate tube contain a large amount of carbon and zinc which indicates the presence of ZIF-8 structure (Fig. 5a).

We also observed that cobalt is only detectable in the silicate template tube underneath the SOD-looking crystals, which also proves that ZIF-8 (Zn(II)–2-MeIm) was formed instead of ZIF-67 (Co(II)–2-MeIm). In order to determine whether SOD or DIA polymorph of ZIF-8 crystals are present on the inner surface of the tube, we performed additional measurements by using SEM and Raman microscopy. The SEM image in Fig. 5b shows the morphology of the formed crystals. They exhibit the characteristic hexagonal shape of sodalite-like ZIF-8 polymorph. To further investigate the product properties, Raman microscopic measurements were also carried out. During our previous work we showed that the main difference between DIA and SOD polymorphs is the vibration bands at around 933 and 759 cm$^{-1}$ corresponding to the out of plane C–H bending and out of plane C=N bending, respectively (Table S1). In case of SOD polymorph, the vibration band is present at 933 cm$^{-1}$ Raman shift, but it is absent at 759 cm$^{-1}$. However, in the case of DIA polymorph, we must experience the opposite^33^. Figure 5c and d show the Raman spectra of the crystals which were present on the inner surface of the template cobalt–silicate precipitate tube. First of all, the spectrum is of high quality and the peaks are well-resolved, thus can be properly interpreted in terms of product polymorph. Furthermore, an intense Raman band appeared at 930 cm$^{-1}$, while no peak is present at 759 cm$^{-1}$ Raman shift. According to this result, the crystals can be identified as the SOD polymorph of ZIF-8, which is in good agreement with the SEM and EDX measurements. In order to further support the presence of the high porosity polymorph (SOD), we attempted to investigate the product properties by powder X-Ray diffraction as well. To carry out these measurements, we had to grind the precipitate tubes in a mortar into a powder. Unfortunately, the characteristic diffractions of SOD did not appear, most probably because they were present in a small quantity among the other components of the tube (e.g. cobalt-silicate). Therefore, we synthesized the functionalized tube again by applying the same protocol as before, but this time 2-MeIm solution was injected for longer time (16 h instead of 3 h) inside the cobalt-silicate tube. Figure 5e shows the background corrected PXRD diffractogram of the sample (raw data is shown in Supporting Information). Clearly, the longer residence time favors the formation of SOD ZIF-8 crystals in larger amount inside the precipitate wall. Although the amorphous character of the sample is dominant—which is in agreement with the known composition of the chemical garden tubes—some diffractions obviously can be assigned. Based on literature data, we identified the product as most probably SOD ZIF-8, which is in agreement with our former results (EDX, SEM, and Raman microscopy). Due to the porous structure of SOD ZIF-8, a high specific surface area of the sample can also indicate its presence. Accordingly, the specific surface area was determined via nitrogen adsorption measurements evaluated by the Brunauer–Emmett–Teller (BET) method. The functionalized tube, which was produced by applying longer injection of 2-MeIm (16 h) exhibits ca. 600 m$^2$/g specific surface area. Although this is much lower than the typical value of a pure SOD ZIF-8 sample synthesized in water ($\sim$1300 m$^2$/g)^33^, keep in mind that our sample mostly contains other components of the precipitate tube (e.g., cobalt-silicate), and it is only decorated with some SOD crystals on the inner surface. As a reference, we also determined the specific surface area of SOD ZIF-8 free (i.e., pristine) cobalt-silicate tube (155 m$^2$/g). The difference between those two values further confirms the presence of SOD crystals in the functionalized tube. To approximate SOD-content of the tube, one can take 1 g sample with 600 m$^2$/g specific surface area. This contains *X* g of SOD with 1300 m$^2$/g specific surface area, and ($1-X$) g of chemical garden tube with 155 m$^2$/g specific surface area. Therefore, equation $1300\,X + 155\,(1-X) = 600$ is obtain, from which *ca*. 35% of the total mass is rendered to SOD crystals, which is not negligible. As it was an obvious conclusion from our PXRD analysis performed on two tube types synthesized over different reaction times (3 h vs. 16 h), the SOD-coverage of the inner tube surface, and thus the amount of SOD produced strongly depends on the reaction time; the longer the time for cross diffusion the more product we obtain inside the tube. Providing more elaborate information on this relationship calls for further investigation. In principle, thermogravimetric analysis (TGA) could provide further insight into the structure and composition of the precipitate tubes. However, as mentioned before, the major component of the precipitate tube is zinc ion precipitated with silicate and/or hydroxide, which might decompose to form oxide as well. This tube is only decorated with SOD ZIF-8 crystals. Therefore, since the background material is highly amorphous, and there might be a zoo of different silicates, we do not anticipate to obtain trustful information from TGA. In addition, producing the appropriate amount of sample is heavily time consuming.

Based on the above results, we presumably produced the SOD polymorph of ZIF-8 crystals on the inner surface of a precipitate tube by reverting the ZnCl_2_ and 2-MeIm solutions, i.e., zinc ions diffuse from outside to inside, which enables the further use of this functionalized tubes. To give an overview on how the synthesis protocol evolved step-by-step, Table S3 in Supporting Information presents a concise but technically still detailed summary of the work.

## Conclusion

In summary, in the newly designed reactor vessel we can synthesize complex and functionalized precipitate tubes which cannot be produced directly. In our work, we used cobalt silicate precipitate tube as a template, and the other reactants were implemented in the system by solution exchanges with the aid of concentration-gradient-driven cross diffusion. During this process, it is important to consider, which of the replacing ions is smaller and therefore more mobile. In order to ensure the diffusion of this ion through the precipitate wall, the concentration ratio between the reactants must be set in such a way that the resulting concentration gradient is favorable. In our case this meant that the more mobile zinc ion had to be placed outside the tube, while the less mobile 2-MeIm reactant was let to flow inside. This way zinc ions diffused into a region, where the other reactant was present in a large stoichiometric excess. These conditions are appropriate for SOD ZIF-8 production^33^, which we most probably observed inside the precipitate tube. Thanks to this new method, which facilitates the production of macroscopic flow-through precipitate pipes, it is possible to supply the traditional chemical garden structures with various additional layers and functions, which is not feasible in case of other, non porous channels (e.g., traditional microfluidic systems), and may pave the way for further applications.

The proof-of-concept experiments we presented here are based on the incorporation of metal–organic frameworks. However, a very similar procedure could be used to decorate the precipitate tube surface with other crystals as well. For example, by functionalizing the precipitate tube with quantum dots, the flow-through pipe could be applied for sensing, as an extension of the idea proposed earlier^22^.

The design of the reactor vessel together with the detailed experimental procedure provided us with ca. 80 % success in growing and functionalizing the precipitate tubes, i.e., only about 20 % of the experiments failed to yield the targeted flow-through precipitate tube. If clogging and air bubble inclusion are avoided, the growth procedure is efficient, and –as presented for the 16 h solution exchange process—the precipitate tubes maintain their connection to the reactor junctions.

## Methods

### Experimental procedure for the production of functionalized flow-through precipitate tube

During our work, we prepared flow-through precipitate tubes in a vertically oriented reactor, and after that we attempted to modify the composition of the tubes by solution exchanges. Different chemicals were used during the experiments: CoCl$_2\,\cdot$ 6H$_2$O, ZnCl$_2$, Na$_2$SiO$_3$, 2-MeIm (C$_4$H$_6$N$_2$), and NaOH. Sodium silicate was available in a solution the composition of which is: (NaOH)$_\text {x}$(Na$_2$SiO$_3$) $\cdot$ zH$_2$O, NaOH: 13.4–14.4%, Si: 12.0–13.0%. According to the nominal composition we calculated the molar concentration of the silicate solution $\approx$ 6 M. Aqueous solutions were used during the experiments. Regarding the experiments, to facilitate appropriate flow directions via buoyant forces, the density of the solutions were also measured (Anton Paar DMA 500 density meter). These data are summarized in Table S2.

**Fig. 6:**
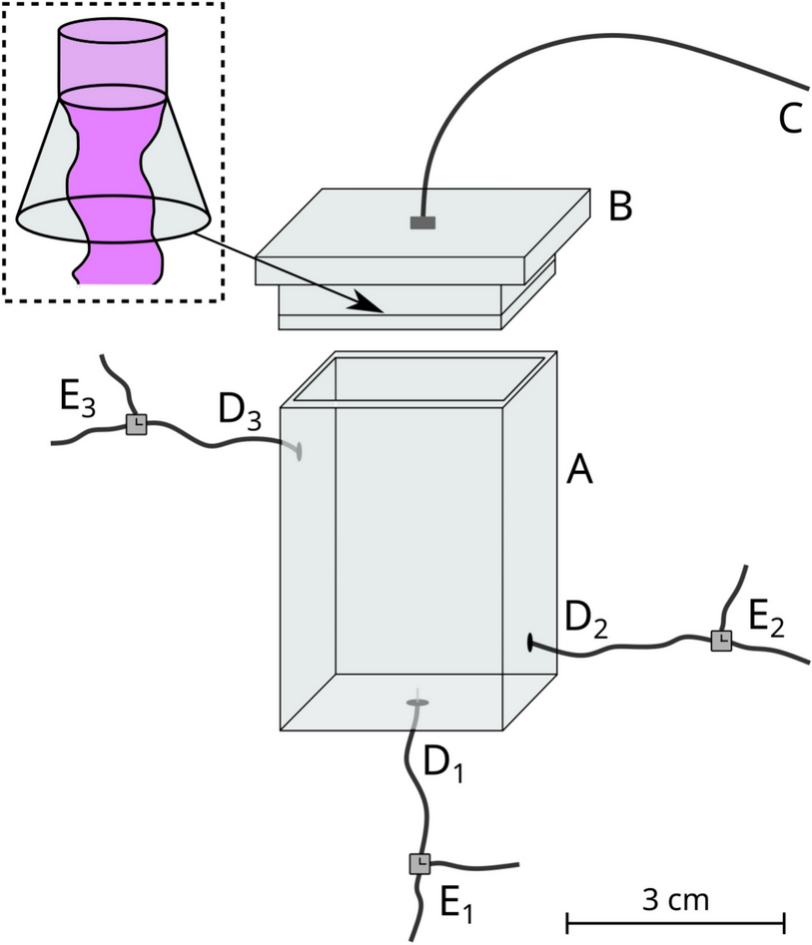
Schematics of the experimental setup: 3-dimensional vertical Plexiglas reactor (description of the notations is included in the main text). The inset shows the conic shape of the cover cup of the reactor which, by its geometry, drives the appropriate precipitate tube growth to achieve reliable and stable junction points.

In order to produce the flow-through precipitate tubes, we designed and built a reactor which was made of Plexiglas (the schematics of this reactor is shown in Fig. 6). The reactor body (*A*) was covered with an airtight upper cup (*B*) which was connected to a tube (*C*) which ensured the outflow of the solutions. Concerning the exchange of the solution in the reactor, we also attached two tubes (*D*$_2$, *D*$_3$) on the two opposite sides of the reactor. During the experiments, one of the reactant solutions was filled into the reactor, and the other reactant solution was injected from below through a needle with a diameter of 0.5 mm. The tubes which were connected to the reactor (*D*$_1$, *D*$_2$, *D*$_3$) contained switchable T-junctions (*E*$_1$, *E*$_2$, *E*$_3$) to change the flow direction of the solutions. In order to ensure the stability and the vertical orientation of the reactor during the experiments, the reactor was placed in a stand and it was fixed at the bottom and top (Fig. S1). The growth of the precipitate tubes was recorded by a digital camera (Adimec-2000m/D) from side-view.

As a first step when setting up the reactor for the experiment, the upper cup was coated with vacuum grease and inserted into its place to close the reactor (Fig. S2a). Afterwards, tube *D*$_1$ was filled with the injecting solution till the tip of the needle (Fig. S2b). In the next step, the host reactant solution was injected manually into the reactor through tube *D*$_2$ by using a plastic syringe (Fig. S2c). The injection of the solution lasted until it passed through tube *D*$_3$ (Fig. S2d). This was necessary to prevent the entry of air bubbles into the reactor during subsequent solution changes. After that, branch *E*$_3$ was closed and the injection of the previous solution continued towards outlet *C*. When the solution went through this spout, the reactor became completely air-free (Fig. S2e). Hereafter, T-junction *E*$_2$ was closed and we opened branch *E*$_1$ towards the reactor. At the end of tube *D*$_1$ the syringe contained the other reactant solution. In addition, a second syringe was located at the end of tube *C*. The two syringes were placed in separate syringe pumps (Fig. S2f). During the experiments, these two syringe pumps were used for the simultaneous injection and withdrawal of the solutions. For the tube growth we applied 1 ml/h flow rate for both pumps. Thanks to this simultaneous injection and withdrawal, a pressure gradient built up along the reactor, which promoted the growth of the precipitate tube upwards. The flow-through character of the obtained precipitate tube was also favored by the inner cone-like design of the reactor upper cup (see the inset in Fig. 6). This was proven by the color of the injected solution which appeared in the outlet tube *C* after the completion of the precipitate tube (see Fig. S2g for schematics, and Fig. S3 for experimental evidence). This required about 25 min in the case of 5 cm reactor height. At this point we simultaneously stopped the syringe pumps.

Once the tube was formed, we attempted to exchange the solutions inside and outside as well. At first the syringe connected to tube *D*$_2$ was removed. It is important to note here that, during the exchange of the external solution we have to take into account the density difference between the solutions. If the density of the replacement solution is lower than that originally filling the reactor, we have to inject the solution through the upper tube (*D*$_3$) and withdraw through the lower tube (*D*$_2$) to avoid buoyancy-driven mixing. If the density distribution is reversed, the injection should be carried out vice versa. During the solution exchange 1 ml/min flow rate was applied. This was significantly higher than what we used for the production of the tube (1 ml/h), but the precipitate tube was strong enough to sustain such conditions. As a result, the solution exchange could be faster. Once the syringe, which contained the replacement solution, was connected to the appropriate fitting (*D*$_2$ or *D*$_3$), the T-junction (*E*$_2$ or *E*$_3$) was closed towards the reactor to pump the air out of the channel (Fig. S2h). After that we started the injection of the solution into the reactor. In parallel with the injection, the withdrawal of the solution was also started (Fig. S2i). This was done until the solution in the reactor was completely replaced (Fig. S2j). In order to illustrate a solution exchange, the replacement solution was colored with a methyl red indicator during the first experiment (Fig. S4).

To exchange the solution inside the precipitate tube, the replacement solution was filled into a new syringe and it was connected to tube *D*$_1$. At first we pumped the air out of the channel, than we started the injection of the solution into the reactor by switching the T-junction *E*$_1$ (at the same time, the withdrawal of this solution was also started through tube *C*), which passes through the precipitate tube (Fig. S2k). The flow rate was set to 1 ml/h. The timescale of the complete solution exchange was varied in case of each experiment. Finally, the precipitate tube was washed inside (45 min) and outside with distilled water in the same way as the solution exchange above, in order to prevent the adsorption of different reactants on the surface, which could interfere with the further measurements (SEM, EDX, Raman microscopy, PXRD, and nitrogen adsorption). The tube was left in distilled water for further 30 min. Afterwards we opened the reactor and took sample from the tube, and let it dry at ambient temperature.

### Microstructure analysis

 The microstructure of the inner and outer surface of the tube was investigated via a scanning electron microscope connected to an energy dispersive X-ray spectroscope (SEM with EDX, Hitachi S-4700). In case of the SEM analysis 10 kV accelerating voltage was applied, but for the EDX measurement this was raised to 20 kV. The samples from the tubes were taken after functionalization, then we let it dry under ambient conditions. Finally, the samples were placed onto conducting carbon tape and were coated with a thin gold layer in order to ensure a proper electrical conductance. We highlight here that the approximated sampling depth of EDX is 2 μm, while the thickness of the precipitate wall is 15–20 μm, thus the registered EDX signal can be validly assigned to the investigated material and does not originate from the underlying carbon tape. The presence of ZIF-8 crystals on the inner surface of the precipitate tube was also confirmed by Raman spectroscopy. Raman spectra were collected with a Bruker Senterra II Raman microscope by using a light source of 785 nm wavelength and 25 mW laser power. Final data were obtained by averaging 16 spectra with an exposition time of 10 seconds. For this measurements the samples were obtained in the same way as in case of SEM analysis. In case of the longer experiments (16 hours 2-MeIm injection), the crystalline phase of the functionalized tube could be also determined by using powder X-ray diffraction (PXRD). The dry precipitate tubes were studied with a Rigaku MiniFlex II Desktop type X-ray diffractometer by applying a CuK$_\alpha$ (= 0.1542 nm) radiation source. The diffractograms were recorded in the 2$\Theta$ = 5–40° range with 0.02° step size. In order to obtain information about the specific surface area of these tubes, we also performed gas adsorption measurements (NOVA3000e, Quantachrome instruments) using nitrogen as adsorbent. To collect the appropriate amount of solid products, the tubes which were formed during the parallel experiments were mixed. These samples were pretreated under vacuum for 6 h at 150°C before the measurements. The specific surface area of the tubes was calculated by BET method on the adsorption band data. Because of the lack of amount of sample required to register whole isotherms, the nitrogen adsorption measurement was only performed between 0 and 0.3 p/p° at 5 equally distributed values (see Supporting Information for details).

## Supplementary Information

Supplementary Information.

## Data Availability

All the data are available on request from the corresponding author.
