# Supplementary material for: Synthesis of metal–organic framework functionalized macroscopic flow-through precipitate tubes
Source: Sci Rep. 2025 Apr 17;15:13241. doi: 10.1038/s41598-025-97630-y (PMC12006425; doi:10.1038/s41598-025-97630-y)
Supplement: Supplementary file 1 — Supplementary Information. [file 41598_2025_97630_MOESM1_ESM.pdf]

Supporting Information: Synthesis of metal organic framework functionalized macroscopic flow through precipitate tubes

Edina Balog, Kinga Bene, and Gábor Schuszter*

Department of Physical Chemistry and Materials Science, University of Szeged, Rerrich Béla tér 1., Szeged, H-6720, Hungary

E-mail: schuszti@chem.u-szeged.hu

Contents

S1 Raman Band Assignment **S3**

S2 Density of reactant solutions **S3**

S3 Experimental setup **S4**

S4 Production of functionalized flow-through precipitate tubes **S5**

S5 Proof of the flow-through character of precipitate tubes **S6**

S6 Confirmation of successful solution exchange **S6**

S7 Raw PXRD data **S6**

S8 Nitrogen adsorption measurements **S8**

S9 Elaboration of the synthesis protocol **S8**

References **S10**

S1 Raman Band Assignment

Table S1: Raman band assignments of ZIF phases.[1–3] SOD polymorph related bands are denoted with $\star$.[4]

Raman shift / cm^{-1}	Band assignment
645	Imidazolate ring puckering
686	Imidazolate ring puckering, H out of plane bending
759, –*	C=N out of plane bending, N–H bending
833	C–H out of plane bending (C4–C5)
945, 933*	C–H out of plane bending (C2–H)
1019	C–H out of plane bending
1143	C5–N stretching
1180	C–N stretching and N–H wagging
1311	ring expansion and N–H wagging
1378	CH_3 bending
1454	C–H wagging
1500	C2N3 and C4N3, C5N1 stretching, and N–H wagging

S2 Density of reactant solutions

Table S2: The density (ρ) of the applied solutions, which determines the location and direction of solution exchanges. *2-MeIm solution contains 0.1 M NaOH as well.

<table>
<tr><th colspan="2">Solutions</th><th colspan="2">c / M</th><th>ρ / $g\,cm^{-3}$</th></tr>
<tr><td colspan="2">$CoCl_2$</td><td colspan="2">0.50</td><td>1.056</td></tr>
<tr><td colspan="2" rowspan="2">$ZnCl_2$</td><td colspan="2">0.05</td><td>1.003</td></tr>
<tr><td colspan="2">0.50</td><td>1.055</td></tr>
<tr><td colspan="2">$C_4H_6N_2$ (2-methylimidazole)</td><td colspan="2">1.00</td><td>1.002</td></tr>
<tr><td rowspan="2">$C_4H_6N_2$</td><td rowspan="2">NaOH*</td><td>1.00</td><td>0.10</td><td>1.010</td></tr>
<tr><td>0.50</td><td>0.10</td><td>1.007</td></tr>
<tr><td colspan="2">Na_2SiO_3</td><td colspan="2">0.75</td><td>1.236</td></tr>
</table>

S3 Experimental setup

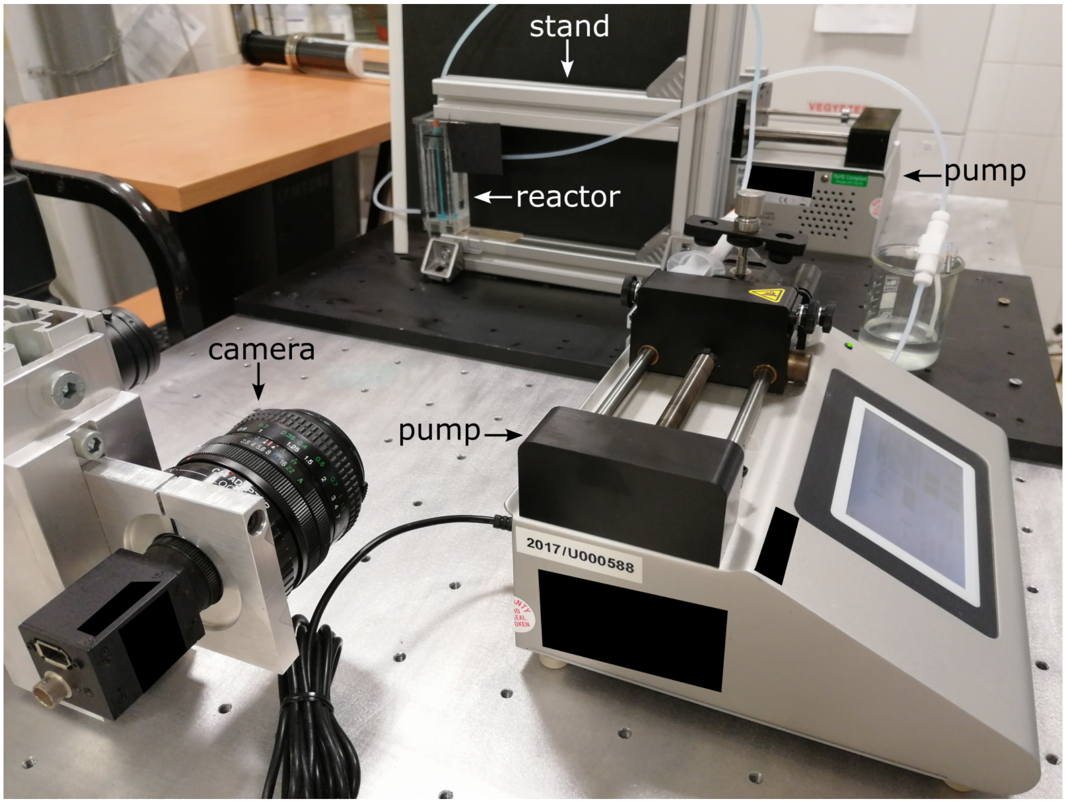

Figure S1: Image of the experimental setup. A blue color flow-through precipitate tube is also shown inside the reactor.

S4 Production of functionalized flow-through precipitate tubes

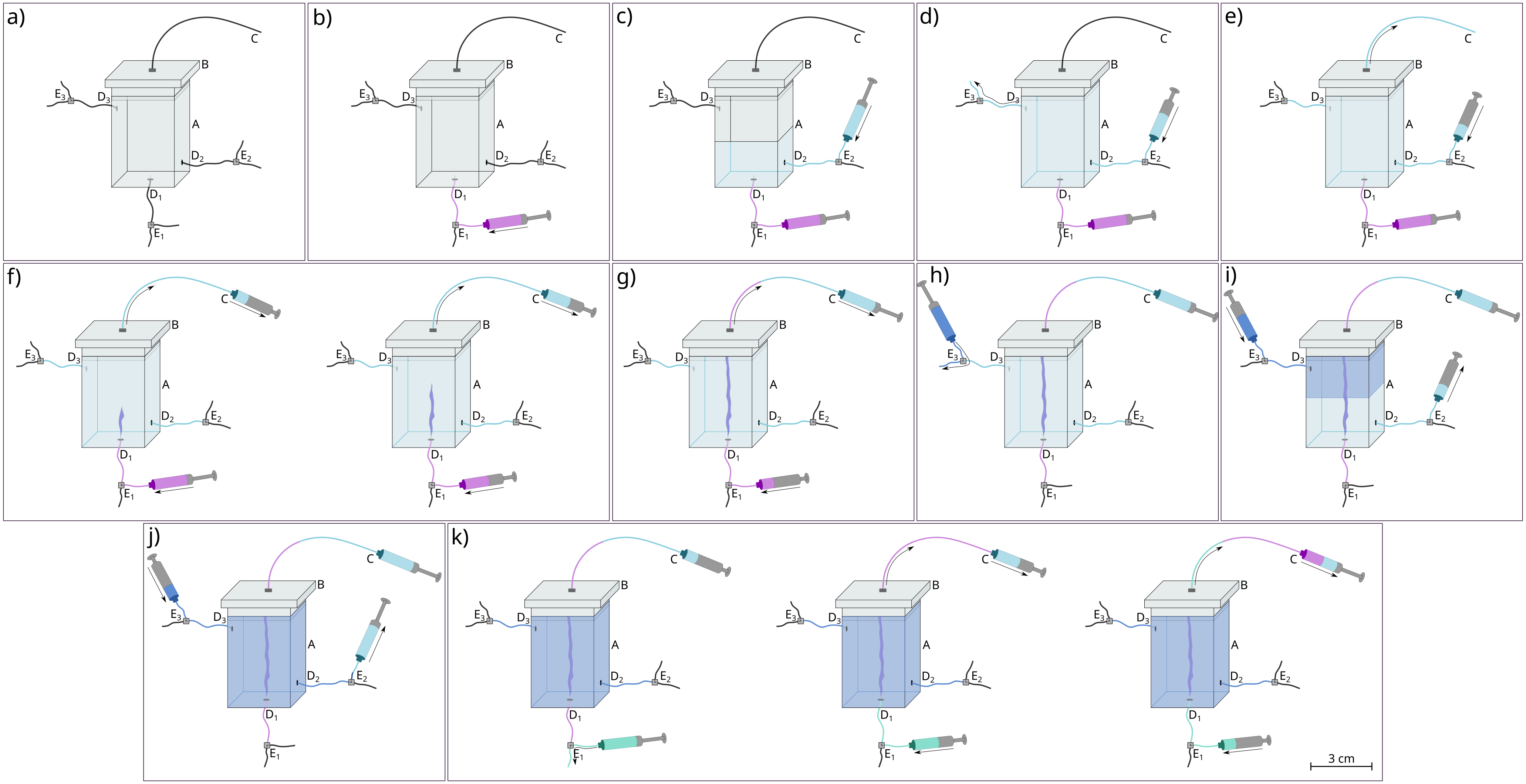

Figure S2: Process diagram for the production of precipitate tubes and for their functionalization by solution exchanges. See the text for step-by-step explanation.

S5 Proof of the flow-through character of precipitate tubes

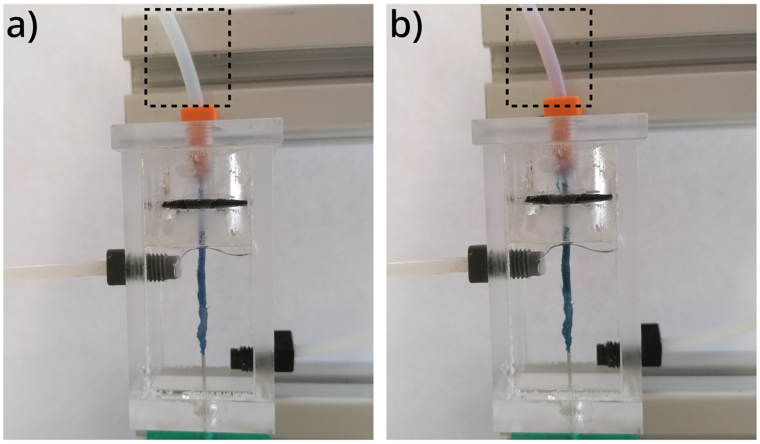

Figure S3: Images showing the flow-through character of the precipitate tubes: **a)** before, and **b)** after the appearance of the injected cobalt-chloride solution in the outlet tube.

S6 Confirmation of successful solution exchange

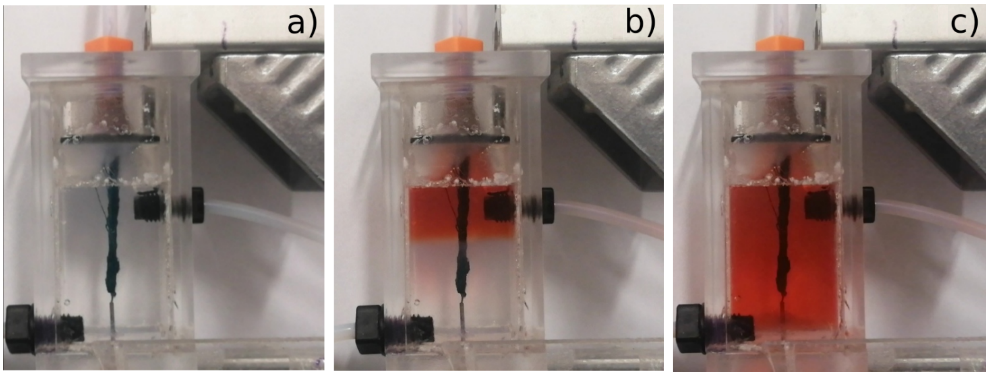

Figure S4: The series of images showing a successful solution exchange: **a)** before, **b)** during, and **c)** after the solution exchange.

S7 Raw PXRD data

As it can be clearly seen, the background gradually increases with 2Θ. This is caused by two reasons. First, the majority of precipitate tube is amorphous as it is composed of various silicates. Second, the template tube contains a large amount of cobalt which is known to result in baseline distortion when measured via standard CuK_α (=0.1542 nm) radiation source. Nevertheless, the characteristic diffractions of SOD ZIF-8 are still visible.

Although the diffractogram does not reach the common level, please consider that a minor crystalline component is sought inside an amorphous precipitate matrix. In the manuscript, we performed background correction in order to suppress the hump caused by the amorphous materials and to better visualize the presence of SOD ZIF-8s.

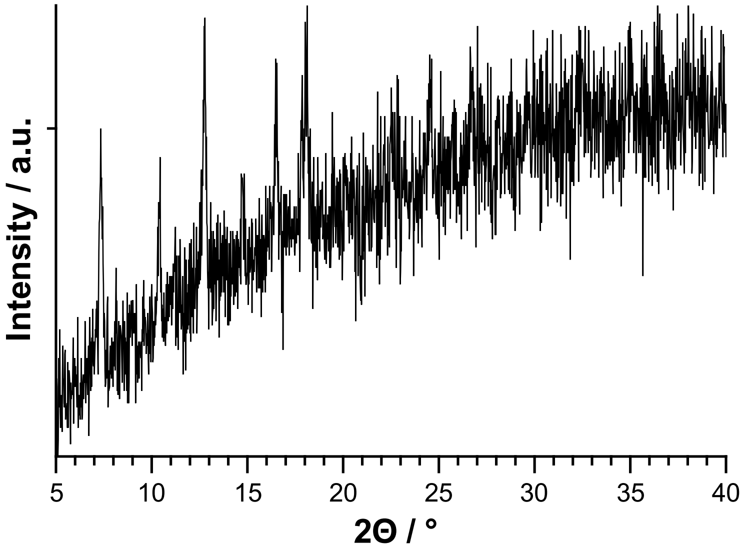

Figure S5: Raw PXRD diffractogram corresponding to Fig.5e in the manuscript.

S8 Nitrogen adsorption measurements

Table S3: Raw data of nitrogen adsorption measurements obtained on the adsorption band and used to determine specific surface area by applying the BET method.

	Relative pressure $\left(\frac{p}{p_0}\right)$	Volume @ STP cm^3/g	$\frac{1}{W((p_0/p)-1)}$ 1/g
Pristine chemical garden tube	0.100059	42.0843	2.1138
	0.165771	45.1937	3.5180
	0.198261	46.4920	4.2558
	0.265752	49.2210	5.8835
	0.300539	50.5906	6.7954
SOD ZIF-8 decorated chemical garden tube	0.117438	213.0539	0.49972
	0.161270	213.7016	0.71990
	0.209116	214.1858	0.98772
	0.256791	214.6714	1.2878
	0.308878	214.1385	1.6699

S9 Elaboration of the synthesis protocol

For an easier technical overview, in Table S4 we show the step-by-step progress for the synthesis of flow-through chemical garden tubes decorated via cross-diffusion.

Table S4: Major steps of the evolving synthesis protocol. See the manuscript for more explanation.

Aim	Injected solution	Host solution	Rate	Time	Solution exchange	Further injection	Result
Direct growth of ZIF-8 tube	0.05 M $ZnCl_2$	1 M 2-MeIm	any	–	–	–	failed ⟶ grow template tube functionalized via cross-diffusion
Functionalized template tube	0.5 M $ZnCl_2$	0.75 M Na_2SiO_3	1 ml/h	45 min	outer solution: 1 M 2-MeIm	4.0 ml/h, 3 h	probably SOD ZIF-8 inside & outside, but possible dissolution of zinc–silicate garden
Template without Zn	0.5 M $CoCl_2$	0.75 M Na_2SiO_3	1 ml/h	60 min	outer solution: 1 M 2-MeIm, inner solution: 0.05 M $ZnCl_2$	0.4 ml/h, 1.5 h	few SOD ZIF-8 outside, many DIA ZIF-8 inside ⟶ no sufficient 2-MeIm influx?
More imidazolate ion	0.5 M $CoCl_2$	0.75 M Na_2SiO_3	1 ml/h	60 min	outer solution: 1 M 2-MeIm containing 0.1 M NaOH for more efficient linker deprotonation; inner solution: 0.05 M $ZnCl_2$	0.4 ml/h, 1.5 h	high [Co^{2+}] in the template, zinc cover inside; SOD ZIF-67 instead of SOD ZIF-8 ⟶ cross diffusion of 2-MeIm hindered by size?
More imidazole inside	0.5 M $CoCl_2$	0.75 M Na_2SiO_3	1 ml/h	60 min	outer solution: 1 M 2-MeIm; inner solution: 0.05 M $ZnCl_2$	– ml/h, 18 h	2-MeIm does not diffuse in
More time for diffusion	0.5 M $CoCl_2$	0.75 M Na_2SiO_3	1 ml/h	60 min	outer solution: 1 M 2-MeIm; inner solution: 0.05 M $ZnCl_2$	0.4 ml/h, 3 h	negligible cross diffusion of 2-MeIm
Let Zn^{2+} ion diffuse in	0.5 M $CoCl_2$	0.75 M Na_2SiO_3	1 ml/h	60 min	outer solution: (⟶water⟶) 0.5 M $ZnCl_2$; inner solution: (⟶water⟶) 0.5 M 2-MeIm containing 0.1 M NaOH for more efficient linker deprotonation	0.4 ml/h, 3 h	template successfully decorated with SOD ZIF-8; no PXRD pattern could be registered ⟶ provide longer time for diffusion
Let Zn^{2+} ion diffuse in	0.5 M $CoCl_2$	0.75 M Na_2SiO_3	1 ml/h	60 min	outer solution: (⟶water⟶) 0.5 M $ZnCl_2$; inner solution: (⟶water⟶) 0.5 M 2-MeIm containing 0.1 M NaOH for more efficient linker deprotonation	0.4 ml/h, 16 h	template successfully decorated with SOD ZIF-8; 3× solution exchange & 16 h injection ⟶ robust precipitate structure; good reproducibility ⟶ proper reactor design

References

(1) Kumari, G., Jayaramulu, K., Maji, T.K. & Narayana, C. Temperature induced structural transformations and gas adsorption in the zeolitic imidazolate framework ZIF-8: a Raman study. *J. Phys. Chem. A*, **117**, 11006 – 11012 (2013).

(2) Carter, D.A. & Pemberton, J.E. Raman spectroscopy and vibrational assignments of 1- and 2-methylimidazole. *J. Raman Spectrosc.*, **28**, 939 – 946 (1997).

(3) Mao, C.J., Hu, X.W., Song, J.M., Niu, H.L. & Zhang, S.Y. Synthesis of zinc 1-(2-pyridylazo)-2-naphthol (Zn(PAN)2) nanobelts with nonlinear optical property. *Cryst. Eng. Comm.*, **14**, 6823 – 6826 (2012).

(4) Balog, E., Varga, G., Kukovecz, Á., Tóth, Á., Horváth, D., Lagzi, I. & Schuszter, G. Polymorph selection of zeolitic imidazolate frameworks via kinetic and thermodynamic control. *Cryst. Growth Des.* **22**, 4268 – 4276 (2022).
